# The impact of age on patient-reported outcomes after oncoplastic versus conventional breast cancer surgery

**DOI:** 10.1007/s10549-021-06126-6

**Published:** 2021-02-19

**Authors:** M. Ritter, B. M. Ling, I. Oberhauser, G. Montagna, L. Zehnpfennig, J. Lévy, S. D. Soysal, L. López Castrezana, M. Müller, F. D. Schwab, C. Kurzeder, M. Haug, W. P. Weber, E. A. Kappos

**Affiliations:** 1 Breast Center, University Hospital of Basel, Spitalstrasse 21, 4031 Basel, Switzerland; 2 University of Basel, Spitalstrasse 21, 4031 Basel, Switzerland; 3 Department of Plastic, Reconstructive, Aesthetic and Hand Surgery, University Hospital of Basel, Basel, Switzerland; 4 Breast Surgery Service, Memorial Sloan Kettering Cancer Center, New York, NY USA; 5 Biometrical Practice BIOP, Basel, Switzerland; 6 Department of Obstetrics and Gynecology, University Hospital of Basel, Basel, Switzerland

**Keywords:** Breast cancer surgery, Oncoplastic surgery, Quality of life, Patient-related outcomes, Age, Elderly

## Abstract

**Purpose:**

Some studies have indicated age-specific differences in quality of life (QoL) among breast cancer (BC) patients. The aim of this study was to compare patient-reported outcomes after conventional and oncoplastic breast surgery in two distinct age groups.

**Methods:**

Patients who underwent oncoplastic and conventional breast surgery for stage I-III BC, between 6/2011–3/2019, were identified from a prospectively maintained database. QoL was prospectively evaluated using the Breast-Q questionnaire. Comparisons were made between women < 60 and ≥ 60 years.

**Results:**

One hundred thirty-three patients were included. Seventy-three of them were ≥ 60 years old. 15 (20.5%) of them received a round-block technique (RB) / oncoplastic breast-conserving surgeries (OBCS), 10 (13.7%) underwent nipple-sparing mastectomies (NSM) with deep inferior epigastric perforator flap (DIEP) reconstruction, 23 (31.5%) underwent conventional breast-conserving surgeries (CBCS), and 25 (34.2%) received total mastectomy (TM). Sixty patients were younger than 60 years, 15 (25%) thereof received RB/OBCS, 22 (36.7%) NSM/DIEP, 17 (28.3%) CBCS, and 6 (10%) TM. Physical well-being chest and psychosocial well-being scores were significantly higher in older women compared to younger patients (88.05 vs 75.10; *p* < 0.001 and 90.46 vs 80.71; *p* = 0.002, respectively). In multivariate linear regression, longer time intervals had a significantly positive effect on the scales Physical Well-being Chest (*p* = 0.014) and Satisfaction with Breasts (*p* = 0.004). No significant results were found concerning different types of surgery.

**Conclusion:**

Our findings indicate that age does have a relevant impact on postoperative QoL. Patient counseling should include age-related considerations, however, age itself cannot be regarded as a contraindication for oncoplastic surgery.

## Introduction

Over the last decades, significant improvements have been made in the treatment of breast cancer (BC), resulting in improved survival rates, especially in early-stage disease [1]. As a result of improved survival outcomes, breast cancer is increasingly perceived as a chronic disease, and thus survivors’ health-related quality of life (QoL) has become a major focus of overall treatment. Since BC can affect different age groups, it seems important to examine age-related differences in terms of QoL. This might contribute to personalized decision making, taking into account this important variable when counseling our patients.

While a vast body of literature examining age-specific differences in QoL related to systemic treatments and survivorship exists [2–4], only few studies have investigated age-related differences in postoperative QoL, comparing different breast surgery procedures [5, 6]. In particular, there is a lack of data on QoL in older patients after BC surgery. This is relevant as a high proportion of newly diagnosed BC affects older women, and evidence is needed to provide proper counseling to this patient population in clinical practice. Previous studies have shown that younger patients have worse QoL outcomes in the social domain, being more concerned with their physical appearance and femininity. Older patients, in contrast, often see their breast appearance as a less important aspect of their QoL, but they tend to score lower in the physical well-being domains [5]. As such, it is important to further investigate possible age-related differences in patient-reported outcomes. Additionally, in the era of personalized BC therapy, age-related differences and requirements should be considered when planning the patient’s treatment [7, 8]. The aim of this study was to compare QoL in two distinct age groups after conventional and oncoplastic breast surgery using the Breast-Q as validated, patient-reported outcome assessment tool [9–11].

## Methods

### Study design and patients

A retrospective review of a prospectively maintained database of consecutive patients undergoing BC surgery between June 2011 and March 2019 by three selected breast surgeons from the Department of Surgery of a tertiary referral center in Switzerland was performed. Women were eligible for study inclusion if they underwent either conventional surgical techniques, such as total mastectomy (TM) and conventional breast-conserving surgeries (CBCS), or oncoplastic breast-conserving surgeries (OBCS), specifically round-block technique (RB) or nipple-sparing mastectomy (NSM) with deep inferior epigastric perforator flap (DIEP) reconstruction. The response rate was 43%.

A cross-sectional survey was conducted using the Breast-Q, a validated, procedure-specific, patient-reported outcome assessment tool, to assess patient satisfaction and health-related QoL via the outcome collecting software Heartbeat® [9–11]. Patients were subdivided into two age groups: < 60 (“younger”) and ≥ 60 years (“older”) according to the age threshold defined by the United Nations [12]. Variables regarding patient, tumor, treatment, and outcome were recorded in a dedicated study database (Secu Trial®).

### Statistical analysis

Patients were analyzed by age and type of surgery: RB/OBCS, NSM with DIEP reconstruction, CBCS and TM. We analyzed the following Breast-Q scales: Physical Well-being Chest, Psychosocial Well-being, Sexual Well-being and Satisfaction with Breasts. Each scale is scored from 0 to 100; higher scores represent more satisfaction or better QoL.

Continuous variables were reported by mean, standard deviation (SD), median, minimum, and maximum values. Categorical variables were summarized by absolute frequencies and percentages. Mean values were compared by age categories using T-tests. Occurrences were compared by age categories using Fisher’s exact test for association. Surgical complications were categorized according to the Clavien-Dindo classification [13]. Recurrence was defined as either local, loco-regional, regional, distant or combined recurrence.

Multivariate linear regression analysis for all Breast-Q scales with age category (< 60 years old, ≥ 60 years old) as covariate was performed. Additional covariates were entered into the model based on stepwise selection: type of surgery (RB/OBCS, NSM/DIEP, CBCS, TM), year of surgery, comorbidities at baseline (yes, no), recurrence (yes, no), time from surgery to follow-up Breast-Q. A p-value < 0.05 was considered statistically significant. All statistical analyses were conducted in SAS version 9.2.

## Results

133 patients operated by breast surgeons with or without plastic surgeons were included in the study. The group of older patients consisted of 73 patients: 15 (20.5%) of them underwent RB/OBCS, 10 (13.7%) NSM with DIEP reconstruction, 23 (31.5%) CBCS, and 25 (34.2%) TM. The younger patient group consisted of 60 women: 15 (25%) underwent RB/OBCS, 22 (36.7%) NSM with DIEP, 17 (28.3%) CBCS, and 6 (10%) TM. In the older patient group the mean age was 71.63 (SD 8.39) years and in the younger patient group 49.72 (SD 7.29) years. The mean time from surgery to follow-up Breast-Q was 37.27 (SD 22.25) months in the older group and 35.34 (SD 25.24) months in the younger group. The groups differed significantly in terms of comorbidities (*p* < 0.001) and type of surgery (*p* = 0.001): older patients had significantly more comorbidities (65.8% vs. 25.0%) and underwent TM more often (34.2% vs. 10.0%); younger patients underwent NSM with DIEP more often (36.7% vs. 13.7%). Additionally, younger patients were significantly more often treated with neoadjuvant chemotherapy (13.3% vs. 2.7%; *p* = 0.042). Other characteristics, such as T-stage, recurrence or surgical complications did not differ significantly (Table 1).

**Table 1:** Patient, tumor and treatment characteristics by age

	Older patients (≥ 60 years) *n* = 73	Younger patients (< 60 years) *n* = 60	*p*-value*
Patient age, mean (SD), yr	71.63 (8.39)	49.72 (7.29)	< 0.001
Mean time from surgery to follow-up Breast-Q (SD), mo	37.27 (22.25)	35.34 (25.24)	0.640
Type of surgery			
RB/OBCS, n (%)	15 (20.5)	15 (25.0)	0.001
NSM with DIEP, n (%)	10 (13.7)	22 (36.7)	
CBCS, n (%)	23 (31.5)	17 (28.3)	
TM, n (%)	25 (34.2)	6 (10.0)	
Unilateral surgery, n (%)	69 (94.5)	52 (86.7)	0.137
Bilateral surgery, n (%)	4 (5.5)	8 (13.3)	
Surgical complications			
No complications, n (%)	56 (76.7)	46 (76.7)	1.000
Clavien-Dindo classification:			
I, n (%)	9 (12.3)	7 (11.7)	
II, n (%)	0 (0)	0 (0)	
IIIa, n (%)	1 (1.4)	1 (1.7)	
IIIb, n (%)	7 (9.6)	6 (10.0)	
IV, n (%)	0 (0)	0 (0)	
V, n (%)	0 (0)	0 (0)	
Neoadjuvant/adjuvant treatment			
Neoadjuvant chemotherapy, n (%)	2 (2.7)	8 (13.3)	0.042
Adjuvant chemotherapy, n (%)	14 (19.2)	11 (18.3)	1.000
Adjuvant endocrine therapy, n (%)	48 (65.8)	39 (65.0)	1.000
Adjuvant radiotherapy, n (%)	41 (56.2)	36 (60.0)	0.488
Preoperative T-Stage**			
cTis, n (%)	8 (11.0)	14 (23.3)	0.244
cT0, n (%)	1 (1.4)	1 (1.7)	
cT1, n (%)	35 (47.9)	26 (43.3)	
cT2, n (%)	24 (32.9)	15 (25.0)	
cT3, n (%)	4 (5.5)	1 (1.7)	
Comorbidities ±			
None, n (%)	25 (34.2)	45 (75.0)	0.000
One or more, n (%)	48 (65.8)	15 (25.0)	
BMI			
Mean BMI (SD)	25.65 (5.06)	24.24 (4.34)	0.090
Recurrence			
Yes, n (%)	2 (2.70)	3 (5.00)	0.657

*Mean values were compared using a T-test. Occurrences were compared using a Fisher's exact test for association

**Missing: 4

± The following comorbidities were assessed at the time of surgery by reviewing the patient records: heart disease, high blood pressure, lung disease, diabetes, liver disease, disease of nervous system, other cancer (within the last 5 years), depression and arthritis

*RB* Round-block technique, *OBCS* Oncoplastic breast-conserving surgery, *NSM* Nipple-sparing mastectomy, *DIEP* Deep Inferior Epigastric Perforator Flap, *CBCS* Conventional breast-conserving surgery, *TM* Total mastectomy

### Physical Well-being chest

**Fig. 1:**
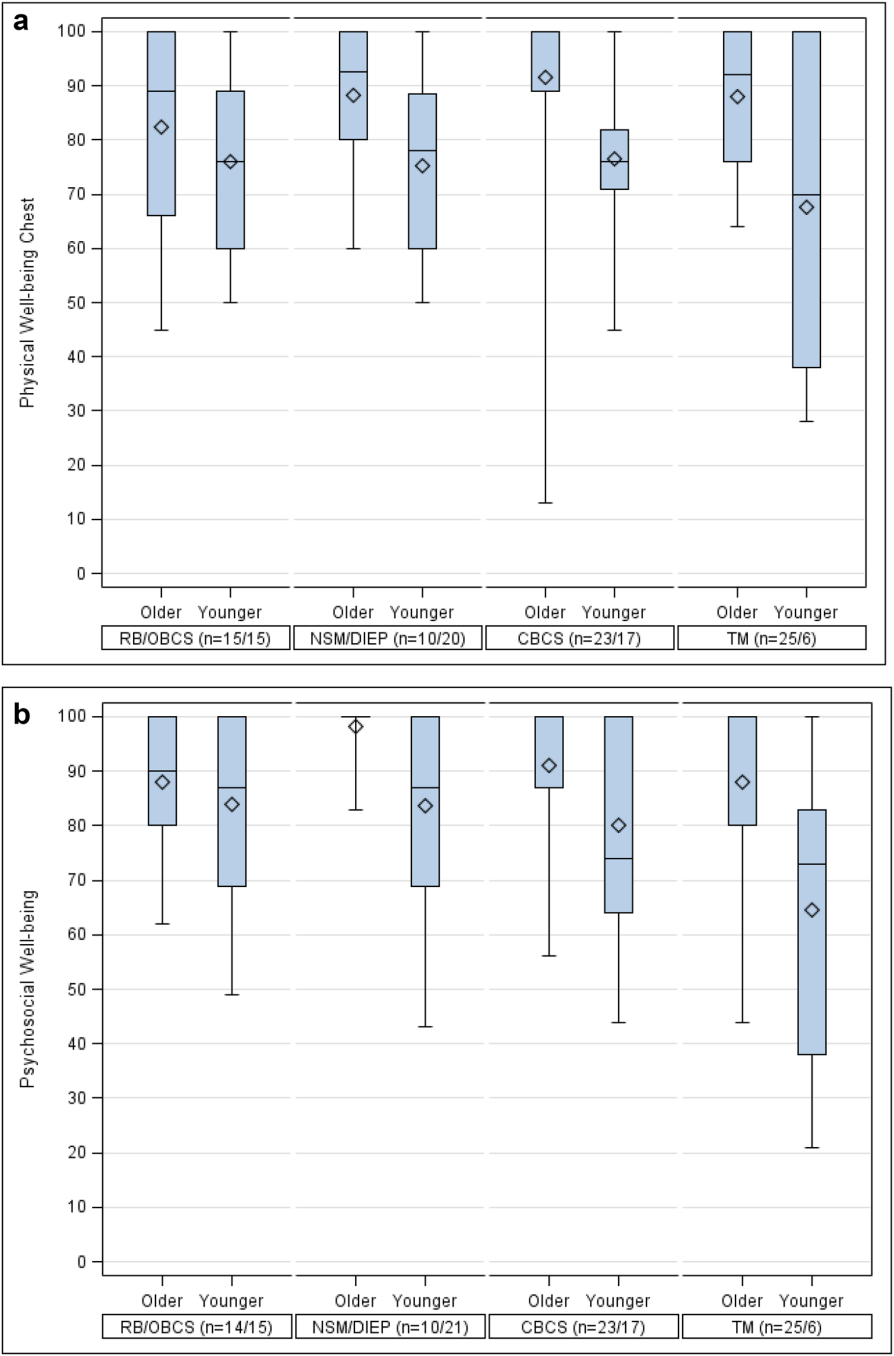

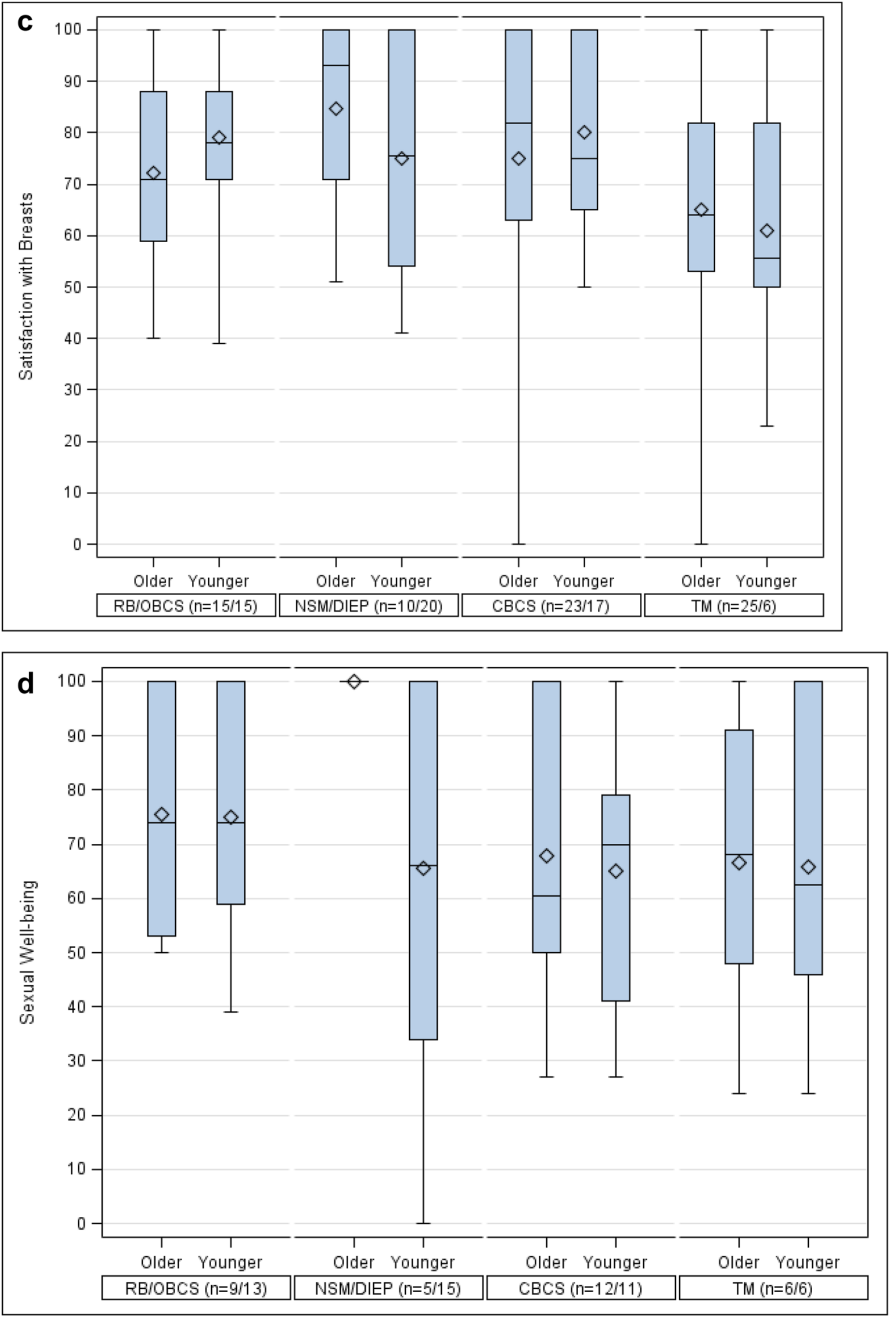
**a** Physical Well-being Chest by age after different procedures, **b** Psychosocial Well-being by age after different procedures **c** Satisfaction with Breasts by age after different procedures, **d** Sexual Well-being by age after different procedures

In older patients, mean scores for physical well-being chest were calculated in all groups: CBCS (91.7 SD 18.7), DIEP with NSM (88.2 SD 14.3), TM (88.1 SD 12.5), and OBCS (82.3 SD 19.2). Regarding the physical well-being of younger patients, mean scores were significantly lower than in older patients (*p* < 0.001). The results were comparable in CBCS (76.7 SD 16.3), OBCS (76.1 SD 15.3) and NSM with DIEP (75.3 SD 18.1), while TM scores were rather lower (67.7 SD 30.9). Overall, however, there were no significant differences between the types of surgery concerning physical well-being chest score (Fig. 1a; Table 2). Multivariate linear regression showed significant effects on the score by age category and by time from surgery to follow-up Breast-Q: better results were found in the older patient group (*p* < 0.001), and results were significantly better with a longer time interval between surgery and Breast-Q survey (*p* = 0.014) (Table 3).

**Table 2:** Age group comparison in different Breast-Q scales

	Older patients (≥ 60 years) *n* = 73	Younger patients (< 60 years) *n* = 60	*p*-value*
Physical Well-being Chest			
n	73	58	
Mean (SD)	88.05 (16.35)	75.10 (18.20)	< 0.001
Min–Max	13.00–100.00	28.00–100.00	
Psychosocial Well-being			
n	72	59	
Mean (SD)	90.46 (14.26)	80.71 (20.48)	0.002
Min–Max	44.00–100.00	21.00–100.00	
Satisfaction with Breasts			
n	73	58	
Mean (SD)	72.30 (22.85)	76.19 (20.69)	0.315
Min–Max	0.00–100.00	23.00–100.00	
Sexual Well-being			
n	32	45	
Mean (SD)	74.75 (24.91)	68.13 (27.09)	0.278
Min–Max	24.00–100.00	0.00–100.00	

*Mean values were compared using a T-test

**Table 3:** Multivariate linear regression analysis for Breast-Q scales

Covariates	Estimate	Standard error	*p*-value
Physical well-being chest: *n* = 131			
Age category			
Younger patients (< 60 years)	0.00		
Older patients (≥ 60 years)	12.75	2.96	< 0.001
Time from surgery to follow-up Breast-Q			
Per month	0.16	0.06	0.014
Psychosocial well-being: *n* = 131			
Age category			
Younger patients (< 60 years)	0.00		
Older patients (≥ 60 years)	9.75	3.04	0.002
Satisfaction with Breasts: *n* = 131			
Age category			
Younger patients (< 60 years)	0.00		
Older patients (≥ 60 years)	− 5.87	3.81	0.126
Year of surgery			
Per additional year	6.30	2.79	0.026
Time from surgery to follow-up Breast-Q			
Per month	0.67	0.23	0.004
Sexual well-being: *n* = 77			
Age category			
Younger patients (< 60 years)	0.00		
Older patients (≥ 60 years)	6.62	6.06	0.279

Age category (< 60 years old, ≥ 60 years old) was included into the model as covariate. Following additional covariates were entered into the model based on stepwise selection: type of surgery (RB/OBCS, NSM/DIEP, CBCS, TM), year of surgery, comorbidities at baseline (yes, no), recurrence (yes, no), time from surgery to follow-up Breast-Q

*RB* Round-block technique, *OBCS* Oncoplastic breast-conserving surgery, *NSM* Nipple-sparing mastectomy, *DIEP* Deep Inferior Epigastric Perforator Flap, *CBCS* Conventional breast-conserving surgery, *TM* Total mastectomy

### Psychosocial well-being

When looking at psychosocial well-being, high values were reported in older patients: NSM with DIEP reached a mean score of 98.3 (SD 5.4), followed by CBCS (91.0 SD 14.1), TM (88.1 SD 17.4), and OBCS (88.1 SD 11.5). In younger patients the mean scores were significantly lower (*p* = 0.002): OBCS (83.9 SD 17.5) and NSM with DIEP (83.6 SD 19.4) scored highest, followed by CBCS (80.1 SD 19.7). TM showed a considerably lower score (64.7 SD 29.8). However, again no significant differences were found among the individual types of surgery (Fig. 1b; Table 2). Multivariate linear regression confirmed significantly better results in the older patient group (*p* = 0.002) (Table 3).

### Satisfaction with breasts

In the older patient group, NSM with DIEP reconstruction achieved the highest scores (84.6 SD 18.3), followed by breast-conserving surgeries, CBCS (75.0 SD 25.3) and OBCS (72.1 DS 18.5). The TM outcome was lower with a mean score of 65.0 (SD 23.0). In the younger patient group, the breast-conserving surgery types, namely CBCS (80.2 SD 19.1) and OBCS (79.2 SD 16.6) showed the highest satisfaction. Comparable results were obtained with NSM with DIEP reconstruction (75.1 SD 22.2). The TM group performed worse with a mean score of 61 (SD 26.9). Once more, differences between the types of surgery were not significant (Fig. 1c; Table 2). Multivariate linear regression showed significant effects on the score by year of surgery and by time from surgery to follow-up Breast-Q: patients surveyed in later years and with a longer time interval between surgery and Breast-Q survey scored significantly better (*p* = 0.026; *p* = 0.004) (Table 3).

### Sexual Well-being

In the older patient group, the questions regarding sexual well-being were answered by 44% (*n* = 32). DIEP with NSM achieved the maximum score (100 SD 0.0), OBCS was rated second (75.6 SD 21.3). The conventional techniques, CBCS (67.8 SD 26.2) and TM (66.5 SD 27.9) showed lower values*.* In the younger patient group, the scale was completed by 75% (*n* = 45): OBCS showed the highest score (74.9 SD 22.4), TM (65.8 SD 30.1), NSM with DIEP (65.5 SD 33.9) and CBCS (65.0 SD 22.1) were rated comparably low. Again, no significant differences between the types of surgery were found (Fig. 1d; Table 2). Multivariate linear regression showed no significant influences on the scores of sexual well-being (Table 3).

## Discussion

This study analyzed patient-reported outcomes in 133 stage I-III BC patients who underwent NSM with DIEP, OBCS, CBCS or TM, comparing older patients, over 60 years of age, with younger patients under the age of 60 years. We have found that older patients achieved significantly higher scores in the psychosocial well-being and physical well-being chest domains compared to younger patients. These findings confirm our hypothesis that there are relevant age-specific differences to be taken into account when informing patients on their surgical options.

### Age group comparison in different Breast-Q domains

Regarding psychosocial well-being, the results are in line with previous research. Studies on BC survivors found that in younger women psychological distress related to diagnosis and treatment and overall psychosocial well-being were significantly worse compared to elderly patients [6, 7]. Concerning physical well-being, on the other hand, there is a discrepancy with the findings reported in literature. Previous studies showed that older women had poorer physical and chest well-being and generally seemed to be more vulnerable to the physical impact of BC regardless of type of surgery [6, 7]. We instead found that older women had better physical well-being scores than their younger counterparts, which is also surprising given the higher comorbidity rate of the older group (Table 1). The good outcome in terms of physical well-being might be explained by the fact that, in our unit, elderly patients are only chosen for oncoplastic surgery if they are in a good general condition for their age. On the other hand, our favorable results among older patients reinforce the concept that age itself should not be considered a contraindication for any surgical procedure.

In our study, we were not able to show any significant difference between the older and younger patients in terms of their sexual well-being score. It has been described in various studies that BC has a stronger negative impact on the sexuality of younger patients [5, 14]. Nevertheless, our results should be interpreted with caution since only 44% of the older patients answered this module.

In terms of breast satisfaction there were comparable overall results in both age groups. One would assume that postoperative breast satisfaction is worse in younger patients, as younger women are more concerned with their physical appearance and femininity [14]. This was not confirmed in our study given the absence of substantial differences in overall scores. This could be associated with the fact that younger patients are more likely to have aesthetically pleasing breasts with less ptosis already preoperatively, whereby the aim is the preservation of aesthetics. In older patients, however, oncoplastic breast surgery can even result in an improvement of aesthetics.

In summary, our study showed significantly higher scores in older patients concerning psychosocial well-being and physical well-being chest compared to younger patients. This suggests that even complex reconstructive surgical techniques could be recommendable for older patients, as they have a very good outcome regarding QoL postoperatively. The literature shows, that there are obstacles to overcome regarding the use of oncoplastic and reconstructive surgery in elderly patients. Among other things, there are prejudices related to body image of older patients, but also insufficient involvement of the patients in the decision making process, when it comes to their surgical therapy and potential breast reconstruction [15]. On the other hand, especially in younger women, care must be taken to become aware of pain or physical limitations in the breast area at an early stage and to treat those problems tiemly. The same applies to psychosocial problems.

### Comparison of satisfaction and QoL by age after different breast procedures

We have found that the results of QoL were more homogeneous in older patients when comparing the four selected types of surgery, without substantial differences after the various procedures. A few comparable studies have shown similar results [16]. The EORTC 10,850 randomized trial, for instance, analyzed survival and QoL in elderly patients, undergoing mastectomy or tumor excision plus tamoxifen. The two groups did not differ in terms of QoL, except that tumor excision patients reported fewer arm problems and a borderline significant benefit in body image [17].

In the group of younger patients, on the other hand, the difference between TM and the other surgical procedures was noticeable in all four domains. The inferiority of mastectomy in terms of quality of life has been shown in several studies, whereas divergent results have been reported when comparing breast-conserving surgeries and SSM/NSM with reconstruction [18–24]. In our study, we could not demonstrate a significant influence of the different types of surgery on the Breast-Q scales. This is most likely due to the too small sample sizes of the individual types of surgery.

### Other influences on Breast-Q scales

In our study, both physical well-being chest and satisfaction with breasts showed significantly better scores with longer time intervals between surgery and Breast-Q survey. Concerning satisfaction with breasts, a study by Atisha et al. showed opposing results [25]. However, the comparability to this study is questionable, as it had a considerably higher mean time interval between surgery and Breast-Q survey of 6.7 years (our study: 3.1 years). Other studies showed a significant increase in breast-Q scores with a longer time interval between surgery and survey [6, 19, 26] or no significant differences in the scores of either physical well-being chest and satisfaction with breasts at different points in time [27, 28]. Even though varying results were found in previous studies, the time interval between surgery and Breast-Q survey seems to be a possible influence on Breast-Q scores, which should be taken into account in future study designs.

Additionally, satisfaction with breasts showed better scores in patients operated on in later years. This can be explained in our particular case by the advanced training of the surgeons and therefore increased use of oncoplastic surgery in later years. A significant influence of the different types of surgery could not be shown directly, which is most likely due to the low sample size per type of surgery.

## Limitations and Strengths

The main limitation of this study is the relatively small sample size. This is due to the fact that we included consecutive patients undergoing a specific set of procedures performed by three senior surgeons during a limited study period since introduction of OPS at the study site. This, in turn, increased comparability of outcomes due to similarities in technical performance of the procedures. Additionally, to make the groups as homogeneous as possible we only included specific types of operations. However, further studies with larger sample sizes are necessary to validate these data.

Another limitation is the considerable variation in the timing of Breast-Q assessment due to the cross-sectional nature of the survey. As mentioned above, previous studies have shown varying results concerning changes of Breast-Q scores over time, suggesting that questionnaires should be collected at comparable postoperative intervals [6, 19, 25–28]. However, the mean time from surgery to follow-up Breast-Q was found to be comparable in both groups.

As this study is a cross-sectional study, there is also no preoperative survey available for comparison, which can also represent a limitation to interpretation of our data.

Finally, this is an observational study, and selection criteria for type of surgery may have an impact on QoL. One strength of our study is that all surgeons in our unit are specialized in oncoplastic surgery, and therefore all surgical procedures are highly standardized. Furthermore, to prevent bias, we specifically looked at the cases of three experienced breast surgeons. Our study provides new insights in a field, where there is still paucity of evidence and our results confirm the relevance of age-specific differences in surgical-related QoL outcomes.

## Conclusion

The current study indicates that age does have an impact on postoperative QoL. Patient counseling should include age-related considerations although age itself should, in our opinion, not be regarded as a contraindication for oncoplastic and reconstructive surgery. It is important not to withhold any surgical techniques from patients based solely on age, since, despite preconceptions, they can have a good or even better postoperative quality of life than younger patients. Our findings support personalized counseling for all women undergoing BC surgery and tailored care to address and anticipate the specific age-related physical and psychosocial needs of these patients. Attention to life-stage issues and concerns can help to improve postoperative QoL and patient satisfaction, as such improving overall patient care. Further studies with larger patient cohorts are necessary to corroborate our findings.
